# Species classification and novel plasmid identifications in *Arcobacter cryaerophilus* and *Arcobacter cryaerophilus-like* organisms

**DOI:** 10.3389/fmicb.2022.984450

**Published:** 2022-09-21

**Authors:** Guilan Zhou, Min Wang, Hairui Wang, Xiaoli Chen, Yixin Gu, Zhujun Shao, Jianzhong Zhang, Maojun Zhang

**Affiliations:** State Key Laboratory for Infectious Disease Prevention and Control, Collaborative Innovation Center for Diagnosis and Treatment of Infectious Diseases, National Institute for Communicable Disease Control and Prevention, Chinese Center for Disease Control and Prevention, Beijing, China

**Keywords:** *Arcobacter cryaerophilus*, genome, taxonomic, phylogenomic analysis, drug resistance, plasmid

## Abstract

The *Arcobacter* is a globally emerging foodborne and zoonotic pathogen that can cause diarrhea in humans. It is relatively homogenous and clearly distinguishes the group from other *Epsilonproteobacteria*. *Arcobacter cryaerophilus* (*A. cryaerophilus*) is a heterogeneous species and little is known about its genomic characterization in China. This study aims to determine the genetic and plasmid features of *A. cryaerophilus* based on whole-genome sequence (WGS). Average Nucleotide Identity (ANI) and *in silico* DNA–DNA hybridization (*is*DDH) were used for the species classification for 90 initially identified *A. cryaerophilus* strains. One complete genome and 42 draft genomes were obtained by whole genome sequencing. The genomic characteristics were determined using various bioinformatics software. The genomes of the strains examined were estimated to vary from 1.81 to 2.28 Mb in length, with a G + C content of around 27%. ANI and *is*DDH results indicated that 90 initially identified *A. cryaerophilus* strains should be reclassified into four new species (ANI > 96% or *is*DDH > 70%). Two clades (four subclades) were identified among 90 genomes with the phylogenetic analysis. The phylogenetic tree indicated these 90 genomes exhibited a high intra-species genomic diversity. No clustering was assorted with the host or geographic location among these genomes. Aminoglycoside resistance genes, such as *aph(2’’)-Ih*, *AAC(6’)-Ie-APH(2’’)-Ia*, *aac(6’)-IIa*, *ant(6)*, and streptothricin resistance gene *SAT-4* were detected in the chromosomes from a third of the Chinese strains. Virulence-related genes were identified in all the sequenced strains. A novel large multiple drug-resistant plasmid (named pCNAC48 with 161,992 bp in length) was identified in strain ICDCAC48. Two antibiotic-resistance islands were found in the plasmid with lengths of 7,950 and 25,137 bp and G + C content of 38.23 and 32.39%, respectively. The drug resistance genes and some transposable elements were cross-distributed among the islands in the plasmid. Antimicrobial susceptibility tests indicated these resistance genes in the plasmid were functional. Plasmid conjugation and curing experiments proved pCNAC48 was stable in strain ICDCAC48. It was the first identified multiple drug resistance plasmid in *A. cryaerophilus-like*.

## Introduction

*Arcobacter* is vastly distributed over various habitats showing to be a large and heterogeneous group accommodating 36 recognized species of diverse origin (Ferreira et al., 2016; Isidro et al., 2020; Parte et al., 2020; On et al., 2021). These species have also gained enhanced attention due to their association with bacteremia in humans, and abortion, mastitis, or diarrhea in animals. *Arcobacter butzleri* (*A. butzleri*) and *Arcobacter cryaerophilus* (*A. cryaerophilus*) are included in the list of microbes that pose a serious risk to human health by the International Commission on Microbiological Specifications for Foods (ICMSF, 2002; Perez-Cataluna et al., 2018a). The disease caused by *Arcobacter* may be self-limited; however, several case studies have reported antibiotic treatment for intestinal and extra-intestinal infections, mainly from the classes of β-lactams, fluoroquinolones, and macrolides (Ferreira et al., 2016; Mottola et al., 2016). Recently, multidrug resistance rates ranging from 20 to 93.8% were reported in *A. cryaerophilus* isolated from different sources, which might affect the treatment of *A. cryaerophilus* infection (Kabeya et al., 2004; Van den Abeele et al., 2016; Rathlavath et al., 2017; Jribi et al., 2020; Sciortino et al., 2021). The actual role of the mechanisms behind antibiotic resistance has not been thoroughly studied, with this lack of information hampering the evaluation of the disease burden of *A. cryaerophilus* and its role as a health hazard.

Perez-Cataluna et al. (2018b) had a taxonomy study for the *Arcobacter* genus based on Average Nucleotide Identity (ANI), *in silico* DNA–DNA Hybridization (*is*DDH), Average Amino-acid Identity, Percentage of Conserved Proteins, and Relative Synonymous Codon Usage, and the study indicated the division of the current *Arcobacter* genus into at least seven different genera (*Arcobacter*, *Aliarcobacter*, *Haloarcobacter*, *Pseudoarcobacter*, *Poseidonibacter*, *Malacobacter*, and Candidate *“Arcomarinus” gen. nov*). According to the results of Perez-Cataluna et al. (2018b), *Arcobacter cryaerophilus* should be called *Aliarcobacter cryaerophilus*. However, On et al. (2020) revealed that the *Arcobacter* genus was relatively homogenous and phylogenetic analyses clearly distinguished the group from other *Epsilonproteobacteria*, and showed that any of the measures used did not support the genomic distinction of the genera proposed by Perez-Cataluna et al. (2018b). In addition, the proposal of Pérez-Cataluña et al. has not been approved by the International Committee on Systematics of Prokaryotes taxonomy subcommittee on *Campylobacter* or validated in the International Journal of Systematic and Evolutionary Microbiology (On, 2021). Two subgroups in *A. cryaerophilus* were once named subgroups 1A and 1B based on DNA–DNA hybridization (Kiehlbauch et al., 1991; Vandamme et al., 1992). Later, Perez-Cataluna et al. described *A. cryaerophilus* as four genomovars based on Multilocus Phylogenetic Analysis, ANI, and *is*DDH but none of the evaluated phenotypic tests enabled their unequivocal differentiation into species (Perez-Cataluna et al., 2018a). These characteristics resulted in the taxonomy difficulties in this species.

Mobile elements, including plasmids, transposons, insert sequences, and integrons, were the most efficient genetic elements promoting the acquisition and dissemination of drug resistance determinants (Vetting et al., 2005; WHO, 2015). One previous report showed that plasmids were present in 9.9% of the *Arcobacter*, but no multidrug-resistant plasmids were reported in *A. cryaerophilus* before (Harrass et al., 1998; Toh et al., 2011; Douidah et al., 2014; Muller et al., 2020). Previous studies have shown that antibiotic resistance of *Arcobacter* might be caused by the resistance genes in chromosomes rather than plasmids (Toh et al., 2011; Douidah et al., 2014; On et al., 2019).

The genomic characteristics of 43 *A. cryaerophilus* strains isolated and identified by PCR in China and 47 genomes obtained from the available database were analyzed in this study. Meanwhile, the species classification for all of the strains previously identified as *A. cryaerophilus* strains was investigated based on the genome sequences. The comparative genetic analysis and the transfer capacities of the novel identified plasmid were also assessed.

## Materials and methods

### Strains and DNA extraction

Forty-three *A. cryaerophilus* strains were isolated from retail raw chicken meat from different districts in Beijing. The strains were identified according to the previous report (Wang et al., 2021) with multiplex PCR and matrix-assisted laser desorption ionization time-of-flight mass spectrometry methods. All identified strains were grown on Karmali agar (OXIOD, United Kingdom) with 5% defibrinated sheep blood as a culture medium. The genomic DNA of each strain was extracted using the QIAamp DNA Mini Kit (Qiagen, Germany) according to the manufacturer’s instructions. The concentration and purity of the DNA were measured using a NanoDrop 2000 (Thermo Fisher Scientific, United States) spectrophotometer.

### Whole-genome sequencing

The genome sequences were accomplished at Beijing Genomics Institute (BGI). Forty-two draft genomes were sequenced using the Illumina HiSeq 2500Xten platform (Illumina Inc., San Diego, CA, United States) generating reads of 300 bp in length and the complete sequencing was performed by PacBio, and long reads were analyzed using the pipelines provided by the SMRT Portal software. Based on the sequence length and alignment method, we discriminated whether the initial assembled sequence was chromosomal or a plasmid sequence and tested whether the sequence was looped. The assembled sequences were predicted as genes and annotated the function using the Prokka pipeline (Seemann, 2014) and RAST annotation server.^1^ RepeatMasker^2^ and TRF software (Benson, 1999) were used to predict scattered repeat sequences and tandem repeats. The amino acid sequence of the plasmid pCNAC48 was compared with the COG, GO, KEGG, NR, and Swiss-Prot databases using BLAST software (Altschul et al., 1990), and its genes and corresponding functional annotation information were combined to obtain the annotation result. Phage Search Tool (PHAST) webserver^3^ and phiSpy software (Akhter et al., 2012) were used to search for phage sequences. The genomic island sequences were predicted using IslandViewer 4 software based on three different genomic islands (GIs) prediction algorithms (IslandPATH-DIMOB, IslandPick, and SIGI-HMM; Bertelli et al., 2017). BLAST Ring Image Generator (BRIG; Alikhan et al., 2011) and Easyfig v2.2.3 (Sullivan et al., 2011) were used for comparative analyses of plasmids and antibiotic resistance genes, respectively.

### Genomes and plasmids of *Arcobacter cryaerophilus* from database

In addition to the strains sequenced in this study, the other genomes of previously assigned *A. cryaerophilus* and plasmids of *Arcobacter* were downloaded from NCBI^4^ and PATRIC^5^ database. SRR data were assembled with SPAdes v3.13.1, and contigs length < 500 bp were filtered. Ninety genomes of *A. cryaerophilus* were selected for this study and genomic information was shown in Supplementary Table S1 (Sheet 1). Other genomes of the *Arcobacter* were downloaded from NCBI and listed in Supplementary Table S1 (Sheet 2). All plasmids used in this study were listed in Supplementary Table S2.

### Species classification and phylogenetic analyses based on the genomes

**Figure 1:**
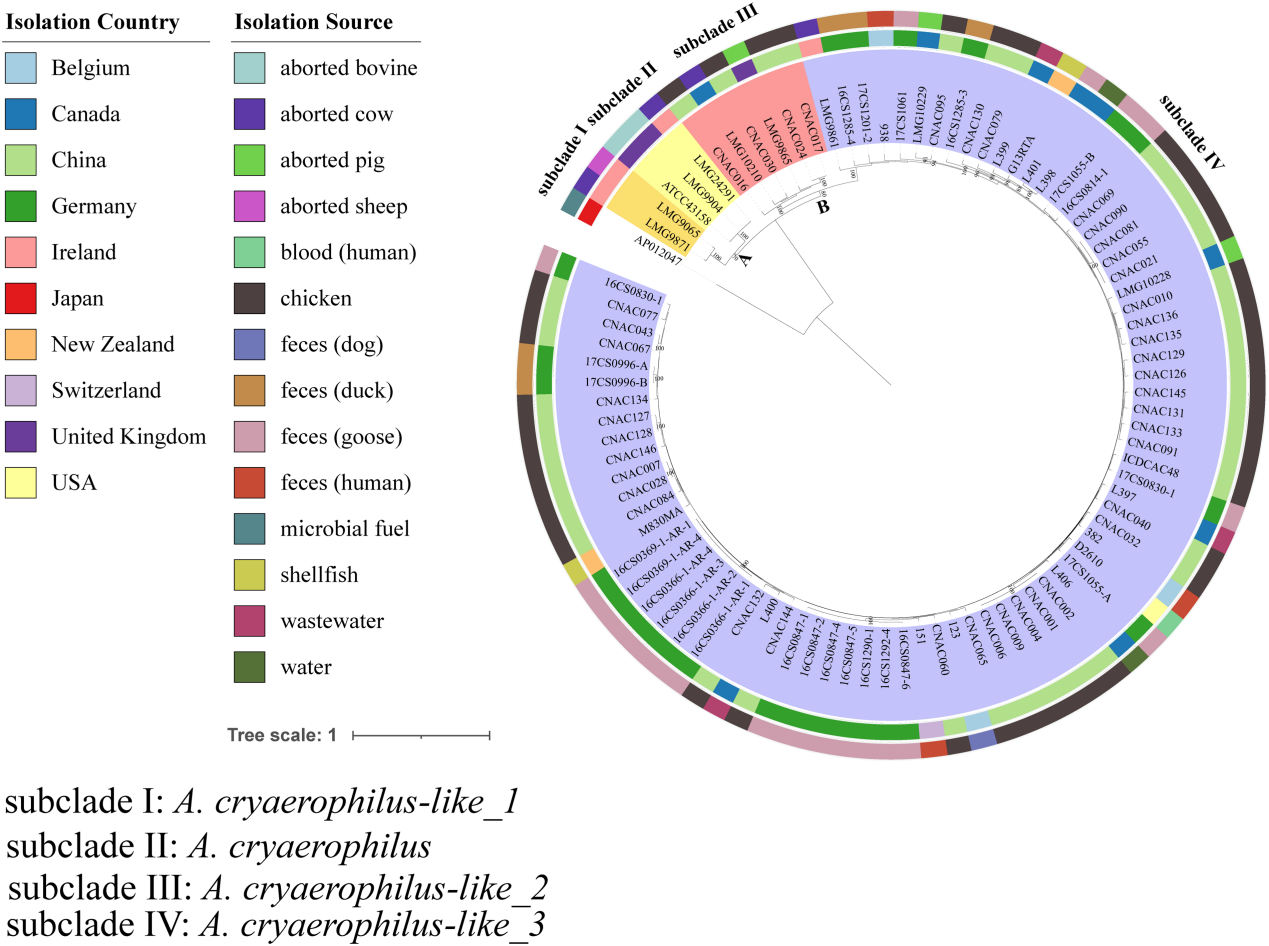
Maximum-Likelihood (ML) tree of the core genome alignments of strains varies from source and country with 1,000 bootstraps. Different colors and shapes indicate various sources. The inner ring represents the strains, the middle ring represents the Isolation Country, and the outside ring represents the Isolation Source. And only bootstrapping values greater than 70 are annotated on the tree. Different background colors indicate different subclades.

16S rRNA sequence of *Arcobacter* was downloaded from the List of Prokaryotic names with Standing in Nomenclature.^6^ The genomes were compared by the ANI (ANIm) and *is*DDH (Formula 2) using pyani^7^ and Genome-to-Genome Distance Calculator software (https://ggdc.dsmz.de; Meier-Kolthoff et al., 2022), respectively. The generally accepted ANI and *is*DDH boundary values for species delineation were 95–96 and 70%, respectively (Kim et al., 2014). Core SNPs were called using Snippy 4.3.6 software^8^ with the genome of a type strain of *A. cryaerophilus* ATCC43158 as a reference, and genome AP012047 (*A. butzleri*) was used as an outgroup. Gubbins software (Croucher et al., 2015) was used to remove the recombination and obtain the pure SNPs without recombination. Phylogeny reconstruction based on core-SNPs was performed with the Maximum-Likelihood (ML) method using MEGA 11 (Tamura et al., 2021) software with 1,000 bootstraps (Figure 1).

### Putative virulence and antibiotic resistance genes screening and antimicrobial susceptibility test for ICDCAC48

Virulence genes of all the genomes were detected on VFanalyzer.^9^ Common virulence genes in *Arcobacter* were identified using BLAST software. The identity cut-off and query coverage values were kept at >90 and >90%, respectively. Resistance genes were predicted using the Abricate^10^ and Comprehensive Antibiotic Resistance Database (CARD; https://card.mcmaster.ca/?q=CARD/ontology/35506), with an E-value of at least 1e−10 as the cutoff. BLAST and mafft (Katoh and Standley, 2013) were used to detect an individual missense mutation in the *gyrA* gene, which was responsible for conferring ciprofloxacin resistance. For ICDCAC48, antibiotic susceptibility testing was carried out with erythromycin, azithromycin, ciprofloxacin, nalidixic acid, tetracycline, gentamicin, florfenicol, telithromycin, streptomycin, chloramphenicol, and clindamycin using gradient strip method (E-test™, bio Mérieux, Nürtingen, Germany) following the manufacturer’s instructions. *C. jejuni* ATCC 33560 was used as a quality control strain.

### Conjugation and plasmid curing assays

The mating experiment was performed to evaluate the conjugation transferability of pCNAC48 among *A. cryaerophilus* strains and some *Campylobacter* strains. The erythromycin-resistant and streptomycin-sensitive strain ICDCAC48 was used as the donor, and the erythromycin-sensitive and streptomycin-resistant *A. cryaerophilus* and *Campylobacter* strains were used as recipients. Eight *A. cryaerophilus* strains and eight *Campylobacter upsaliensis* strains were selected for the assessment in this study. A donor and recipient mixture with a concentration ratio of 1:9 was grown in BHI broth at 37°C overnight. One hundred microliters of each mixed culture were spread on a Karmali medium with 100 μg/ml erythromycin and 50 μg/ml streptomycin for 3 days of incubation. Then, the potential transconjugants which might obtain the resistant plasmid were selected from the selective medium and confirmed by PCR amplification of the specific genes in the pCNAC48 plasmid. The primers designed for specific sequences in the plasmid were listed in Supplementary Table S3. The mating experiment for each tested strain was repeated three times.

The stability of the pCNAC48 in ICDCAC48 was evaluated by daily serial passage on an antibiotic-free Karmali medium. Colonies were tested daily for antibiotic resistance genes in pCNAC48 by PCR. One hundred and fifty passages were assessed in this study.

## Results

### Genomic characteristics

Hybrid sequence assembly of the Illumina paired-end reads and PacBio long reads resulted in a single contig representing one chromosome and one circular plasmid in strain ICDCAC48. A total sequence length of 2,211,108 bp with a G + C content of 27.48% in the chromosome, and a putative plasmid was identified with 161,992 bp in length and a G + C content of 27.38%. Following Illumina draft genome sequencing, approximately 100 to 150× reads coverage was obtained for the other 42 genomes. Genome sizes of the examined 42 strains were each in a region of ~2.1 Mb in size. A total of 90 *A. cryaerophilus* genomes (43 sequenced in this study and 47 public database available genomes) were subjected to deep comparative analysis. Based on these 90 genomes, the chromosome of *A. cryaerophilus* was estimated to vary from 1.81 to 2.28 Mb in length, with an average G + C content of around 27%. The comprehensive genomic characteristics of the sequenced strains were listed in Supplementary Table S1 (Sheet 1).

### Species classification and genetic population

The ANI and *is*DDH values of 27 represent type genomes of the *Arcobacter* genus were listed in Supplementary Table S4 (Sheet 1). High ANI and *is*DDH values were found among some species, such as the ANI and *is*DDH values between *A. marinus* and *A. canalis* were 95.67 and 63.7%, respectively. According to the ANI (>96%) and *is*DDH(>70%), these 90 *A. cryaerophilus* genomes in this study were classified as four different species. Within these four species, ANI values were >96%, *is*DDH values of almost all genomes were >70%, with the exception of some genomes (16CS1285-4, 938, 17CS1201-2, and LMG9861), but these *is*DDH values were >68%. ANI and *is*DDH values were <95 and <60% among different subclades, except for a few strains in subclade III and subclade IV, which reached 95 and 60%, respectively. The results from the calculated overall genome-related taxonomical indices ANI and *is*DDH were shown in Supplementary Table S4 (Sheet 2) and Table 1. Type strain ATCC43158 of *A. cryaerophilus* was allocated in subclade II. Strains from China were identified as *A. cryaerophilus* by PCR but distributed in subclade III and subclade IV, named *A. cryaerophilus-like_2* and *A. cryaerophilus-like_3*. An ML tree was constructed using the core genome alignment of 90 strains to analyze the resulting population structure (Figure 1). Two major clades designated clades A and B were identified in these 90 strains. Clade A was subdivided into two subclades named subclade I and subclade II, which were composed of 5 (5.56%) strains isolated from aborted bovine, aborted cow, and aborted sheep in the United Kingdom and Ireland. Clade B was subdivided into two subclades named subclade III and subclade IV. Clade B was the most prevalent, accounting for 94.44% (85/90) and strains from different sources were scatted and distributed among the phylogenetic groups. All strains from China were distributed in clade B. The classified groups in the phylogenies tree were consistent with the classifications according to the ANI and *is*DDH analysis. No clustering of host or geographic location was observed in the strains examined.

**Table 1:** Summaries of the ANIm and *is*DDH between the four genetic subclades of 90 analyzed strains.

	Subclade I	Subclade II	Subclade III	Subclade IV
Subclade I	**98.13–100%** (81.40–100%)	56.10–56.90%	51.00–51.80%	48.4–50.5%
Subclade II	**94.36–94.43%**	**100–100%** (100–100%)	52.40–54.4%	48.40–50.10%
Subclade III	**93.37–93.52%**	**93.73–94.06%**	**96.71–99.99%** (69.70–100%)	58.0–64.10%
Subclade IV	**92.81–93.28%**	**92.74–93.21%**	**94.92–95.88%**	**96.50–100%** (68.20–100%)

Values range (minimum value to maximum value) corresponds to ANIm (bold) and isDDH.

**Figure 2:**
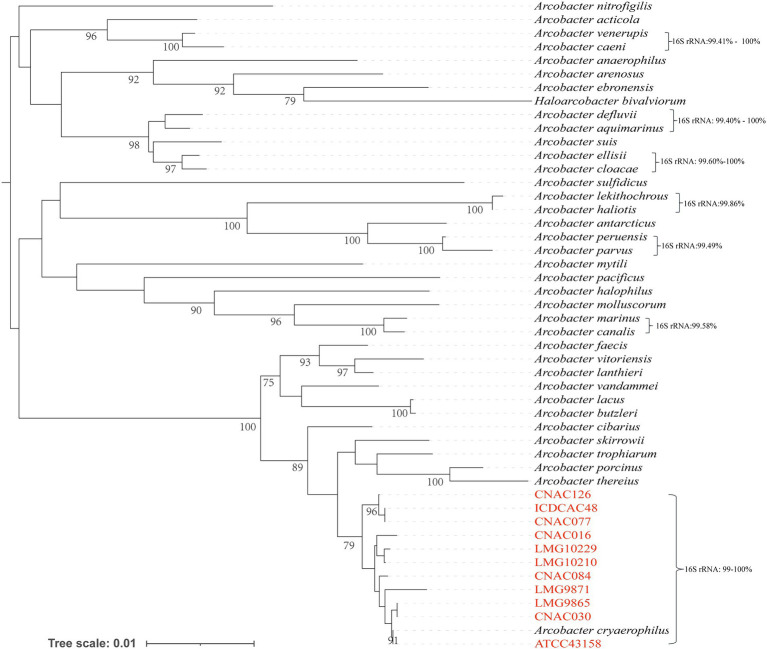
Neighbor-joining tree based on 16S rRNA sequences showing the phylogenetic position of *Arcobacter* and the representative strains of the four subclades. And only bootstrapping values greater than 70 are annotated on the tree. Bar indicated six substitutions per 1,000 bp.

The sequence similarity of the 16S rRNA gene between the type strain (ATCC43158) and the representative strains isolated in this study ranged from 99.14 to 100% (Supplementary Table S5), which was higher than that of other *Arcobacter* species of *Arcobacter* genus. Their close relationship was also visible in the 16S rRNA phylogenetic tree as they were clustered together (Figure 2).

### Virulence factors and antibiotic resistance genes

**Figure 3:**
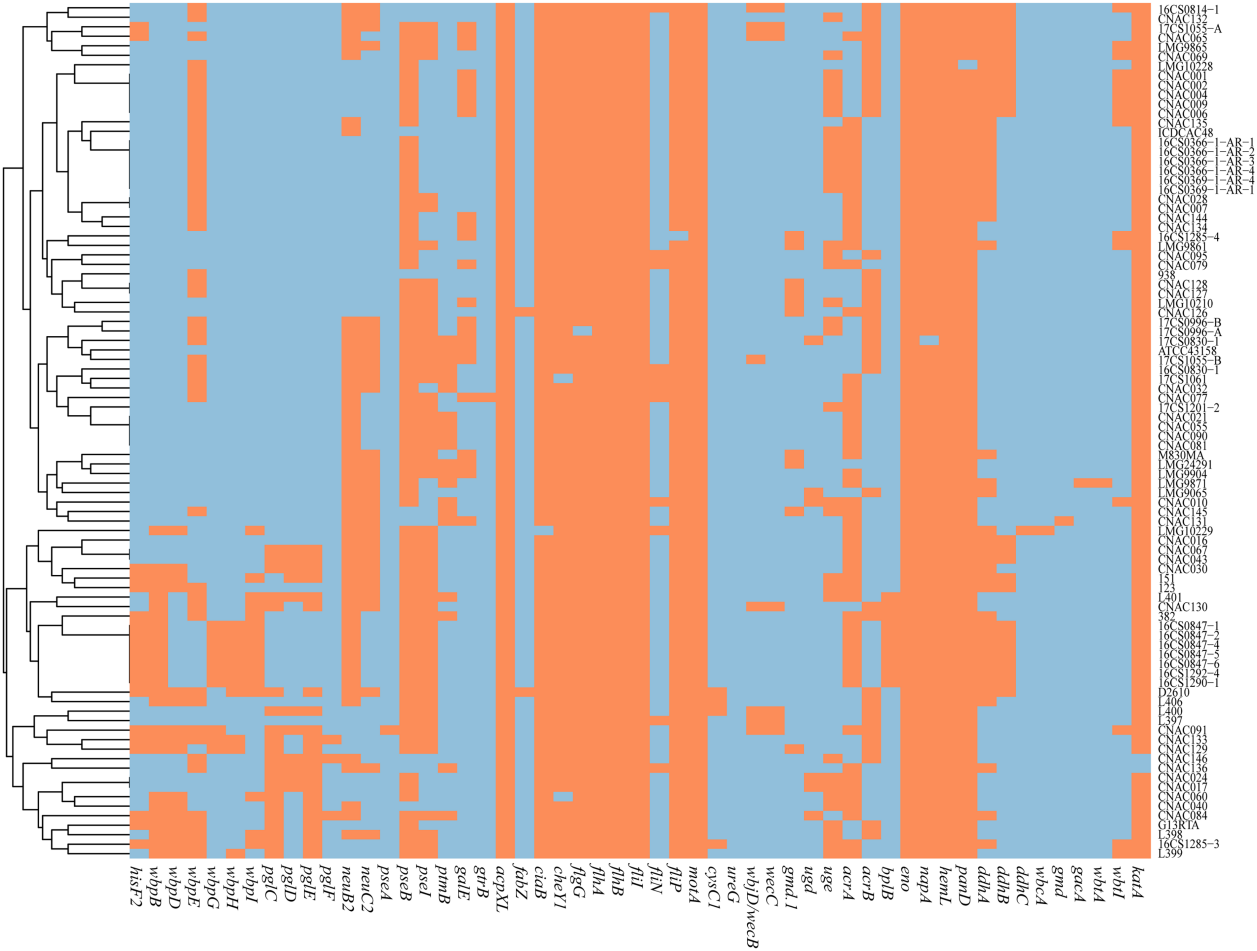
Heatmap is generated using the pheatmap package based on the distribution of virulence genes. Orange indicates the presence of the virulence genes, blue–gray indicates the absence of the virulence genes.

Numerous virulence-associated genes were identified, such as the adhesion-related genes, immune-related genes, motility-related genes, and stress adaptation-related genes (Figure 3; Supplementary Table S6). The *ciaB* (invasion protein), *tlyA* (hemolysin TlyA), *mviN* (peptidoglycan biosynthesis), and *pldA* (phospholipase) were commonly present in all tested strains. The *cadF* (fibronectin-binding proteins) was presented in most tested strains at 97.78% (88/90), while other genes like *hecA* (filamentous hemagglutinin family), *hecB* (hemolysin activator protein HecB), *iroE*, and *irgA* (iron-regulating outer membrane protein) were only detected in some strains. No regional or host origin differences were found in the distribution of virulence genes.

The prediction of resistome by CARD and Abricate showed that aminoglycoside antibiotic resistance genes [*aph*(*2″*)*-Ih*, *AAC*(*6′*)*-Ie-APH*(*2″*)*-Ia*, *aac*(*6′*)*-IIa*, and *ant*(*6*)] were found in 13 of 43 Chinese strains (CNAC007, CNAC016, CNAC028, CNAC040, CNAC043, CNAC065, CNAC067, CNAC069, CNAC091, CNAC095, CNAC144, CNAC145, and ICDCAC48). *SAT-4* was found in CNAC016, CNAC043, CNAC067, CNAC091, CNAC095, CNAC144, and CNAC145. Resistant genes *VEB-3*, *optrA*, *mexA*, *mexB*, *oprM*, *mefA*, *msrD*, and *ere*(*B*) were only identified in strain ICDCAC48 which were harbored in the plasmid pCNAC48.

The search for antimicrobial resistance determinants revealed that all strains from China were susceptible to erythromycin, except for ICDCAC48. The quinolone resistance determining region (QRDR) at position 254 of the *gyrA* gene was found in 55.81% (24/43) strains, including ICDCAC48. The MICs of ICDCAC48 to erythromycin, azithromycin, ciprofloxacin, nalidixic acid, tetracycline, gentamicin, florfenicol, and telithromycin were 64, 32, 32, >64, 32, 16, 8, and 32 μl/ml, respectively. It was sensitive to streptomycin, chloramphenicol, and clindamycin with MICs of 4, 8, and 1 μl/ml, respectively.

### Genetic structure of drug-resistant plasmids pCNAC48 and comparative genomics of *Arcobacter* plasmids

**Figure 4:**
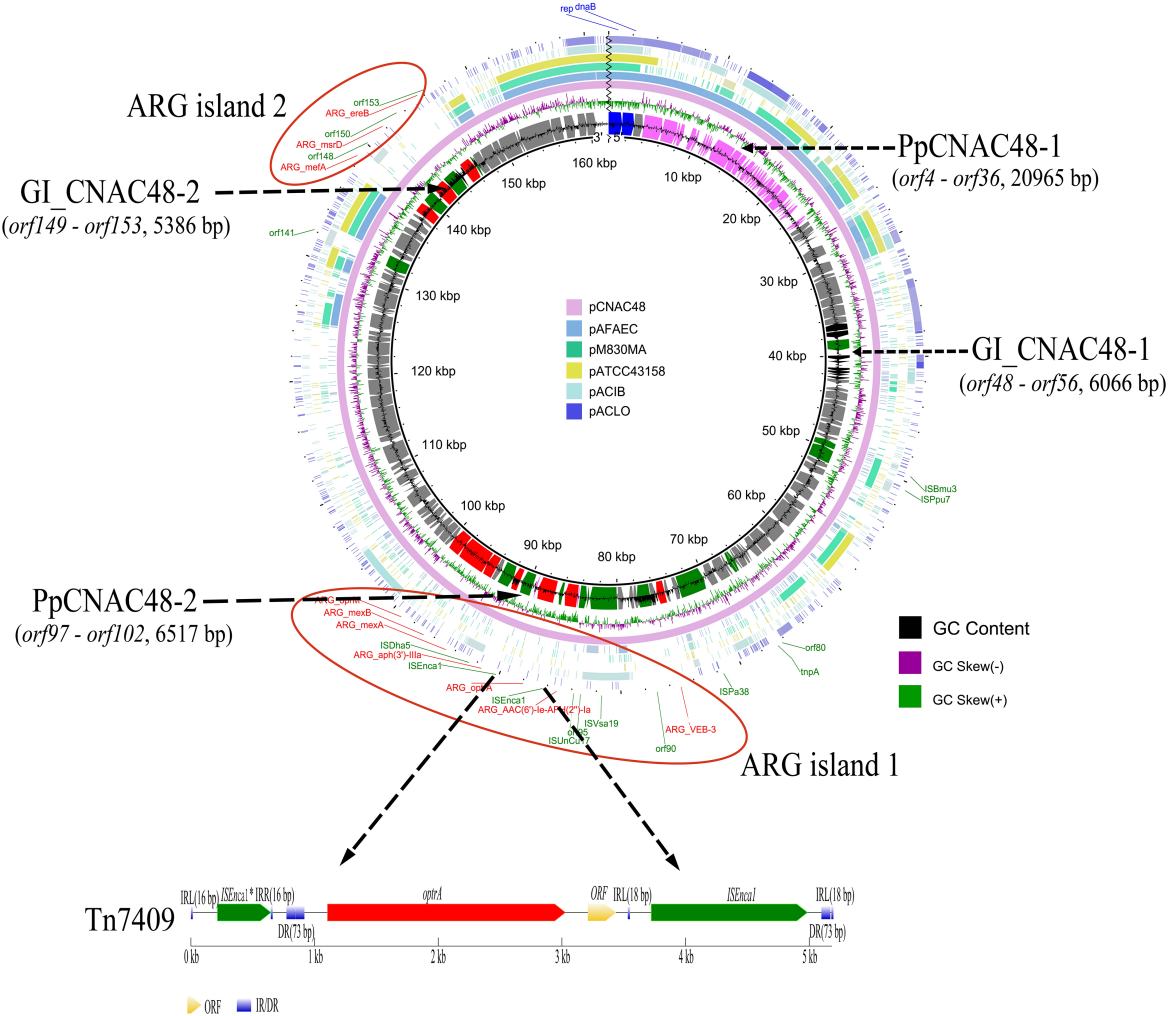
Schematic of the circularized plasmid sequence and position of the gene clusters associated with putative resistance genes. From inside to outside the ring: the GC Content, GC Skew, pCNAC48, pAFAEC, pM830MA, pATCC43158, pACIB, and pACLO, respectively. Different colors represent different genes, blue represents replication initiation protein, red represents antimicrobial resistance genes, green represents transposable associated genes, purple represents prophage genes, and black represents genomic island genes. The red circle represented the drug resistance gene islands. The arrow’s length and direction indicate the gene’s size and direction. Below is the gene environment of Tn7409. IRL and IRR indicate inverted repeats left and right, respectively. DR indicates direct repeat.

One hundred sixty-three genes were identified in pCNAC48, 62 of which were not associated with any known function. The location and the characteristics of ORFs and annotated functions of plasmid pCNAC48 were shown in Supplementary Table S7. The genomic features of comparative plasmids were presented in Figure 4. The backbone of pCNAC48 was closely related to previously identified *Arcobacter* plasmids pM830MA, pATCC43158, and pAFAEC. These plasmids were composed of plasmid replication (1–2,849 bp) and plasmid stability maintenance (151,970–160,575 bp), which were conserved to a certain extent. These plasmids contained the same replicon *repA*, which could not be assigned to any known incompatibility groups (Figure 4). Differences were also found in these plasmids, especially pCNAC48.

The whole plasmid pCNAC48 had a GC content of 27.04%, which was similar to the overall GC ratio (27.49%) in the chromosome of *A. cryaerophilus* (~27%). Two antibiotic-resistance gene islands (*ereB—mefA* and *VEB-3—oprM*) were found in the plasmid with lengths of 7,950 and 25,137 bp and GC content of 38.23 and 32.39%, respectively. Insertion sequence IS1380 family and Tn7409 were found in the plasmid pCNAC48. A truncated copy of *ISEnca1*, named ISEnca1* (a paired terminal 16 bp inverted repeat IRL 5′-TATTATAAAAGACATT-3′ and IRR 5′-AATGTCTGGTATAATA-3′) was present upstream of *optrA*. A paired terminal 18 bp inverted repeat (IRL 5′-TATACCTAGATTCTACGT-3′ and IRR 5′-ACGTGGAATTTAGGAATA-3′) were identified in *ISEnca1* (IS1380 family). Tn7409 was composed of ISEnca1*, *ISEnca1*, *optrA*, and an unknown function gene. The location and genetic characteristics of this transposon were present in Figure 4.

Two genomic islands, named GI_CNAC48-1 (*ORF48 - ORF56*) and GI_CNAC48-2 (*ORF149-ORF153*), were identified in pCNAC48. Sequence analysis showed that the length of the two genomic islands were 6,066 bp (36,425–42,490 bp) and 5,386 bp (141,098–146,483 bp) with the G + C content of 24.15 and 41.53%, respectively. The sequences of genomic islands were compared with those of known microorganisms in the NCBI database, and the coverage rate and identity were low.

Two prophages (complete prophage and incomplete prophage) were identified in pCNAC48, which were named PpCNAC48-1 and PpCNAC48-2. The size of the two prophages were 20,965 bp (3,938–24,902 bp) and 6,517 bp (84,385–90,901 bp) with the average G + C content of 24.59 and 31.15%, respectively. Sequence analysis showed that PpCNAC48-1 had 93.00% query cover and 95.71% sequence similarities with the *Aliarcobacter feces* strain CCUG 66484 plasmid pAFAEC (CP053838.1).

### Transferability of plasmids pCNAC48

No conjugated colony was found from the repeated attempts of mating experiments and none of the resistant lost colonies was identified during the passages study. These results indicated the stability of pCNAC48 in strain ICDCAC48.

## Discussion

*Arcobacter cryaerophilus* is a globally emerging foodborne and zoonotic pathogen with a wide range of sources and regional (Ferreira et al., 2016; Muller et al., 2020). The genomic and classification characteristics were valuable for further investigation of this pathogen. To obtain the genetic and taxonomic characteristics of *A. cryaerophilus*, 90 genomes, including 47 publicly available genomes and 43 newly sequenced strains from our collection, were selected in this study. The quality of the genome sequences was generally in agreement with the minimum standards established for using genome data for taxonomical purposes. Globally, the genomic characteristics of the 90 compared genomes shown in Supplementary Table S1 were very similar, with sizes that did not differ by more than 0.47 Mb, a G + C content ranging from 27.01 to 28.18%, and coding sequences or CDS around 2,100.

The classification of the *Arcobacter* is controversial. Perez-Cataluna et al. suggested that the current *Arcobacter* should be redivided into seven distinct genera, but this proposal has been robustly refuted (On et al., 2020; On, 2021). However, strains identified as *A. cryaerophilus* were without question diverse. Type strain ATCC43158 of *A. cryaerophilus* and representative strains from each subclade (I–IV) were selected for constructing a 16S rRNA gene phylogenetic tree. The percentage of similarity of the 16S rRNA gene between these strains ranged from 99.14 to 100%. These results agreed with what occurred between other species of the *Arcobacter*, where the 16S rRNA gene did not have enough resolution to differentiate the species. However, 16S rRNA phylogenetic tree showed that representative strains in four subclades were genetically closer to *A. cryaerophilus.* For *A. cryaerophilus*, ANI values above 96% were the ones that better correlated with *is*DDH results above 70% in previous studies (Perez-Cataluna et al., 2018a), but this study only concluded based on 13 *A. cryyaerophilus* genomes. Supplementary Table S4 and Table 1 showed the results from the calculated overall genome-related taxonomical indices, i.e., ANI and *is*DDH. The ANI values of the genomes within the subclade were all >96%, while the ANI values from each of the four different subclades were <96%, indicating that these subclades belonged to different species. The *is*DDH among genomes within each subclade was >68% and the *is*DDH among four subclades was <64.1%, indicating that each subclade represented an independent species, which was consistent with ANI. And these results were well demonstrated by other species of *Arcobacter* at the genomic level. For example, the ANI and *is*DDH values of *A. aquimarinus* (GenBank: CP030944) and *A. cloacae* (GenBank: NXII01000000) were 93.31 and 50.9% respectively, and *A. thereius* (GenBank: CP035926) and *A. porcinus* (GenBank: CP036246) were 93.44 and 50.8%, respectively, and *A. marinus* (GenBank: CP032101) and *A. canalis* (GenBank: CP042812) were 95.67 and 63.7%, respectively. The results of taxonomic analysis of 90 genomes of strains initially identified as *A. cryaerophilus* indicated that these strains could be divided into four genomovars, which was consistent with the results of Perez-Cataluna (Perez-Cataluna et al., 2018a). Therefore, we named these strains that were genetically closest to *A. cryaerophilus* but not belonging to *A. cryaerophilus* as *A. cryaerophilus-like*. Our results showed that strains from China belonged to subclades III and IV, also named *A. cryaerophilus-like_2* and *A. cryaerophilus-like_3.* All the strains sequenced in this study were those initially identified as *A. cryaerophilus* by PCR. However, the genomic analysis showed that the PCR method did not have enough discrimination to distinguish these new subclades, which were correctly described as *A. cryaerophilus-like*, although they were similar but not *A. cryaerophilus*. Therefore, specific primers for distinguishing *A. cryaerophilus* and *A. cryaerophilus-like* should be designed in future work. The core SNPs phylogenetic tree showed that the genomes were divided into four subclades with well-supported clusters with bootstraps. The subclade I and subclade III formed a separate branch from subclade II and subclade IV, respectively, which was consistent with the higher values observed with ANI and *is*DDH for these two subclades. Phylogenetic analysis showed the strains examined exhibited a high intraspecies genomic diversity, which may reflect the high adaptability to host and environment. However, clade B was the most prevalent, accounting for 94.44% (85/90), and all strains from China were distributed in clade B. Notably, strains recovered from human specimens belonged exclusively to clade B, suggesting potential host specificity.

*Arcobacter cryaerophilus* is considered an emerging zoonotic pathogen, and available data on virulence and antibiotic resistance genes are still limited. In this study, the adhesion-related genes, immune-related genes, motility-related genes, and stress adaptation-related genes were identified in almost all strains. Interestingly, we observed that *cadF*, *ciaB*, *mviN*, *pldA,* and *tlyA* were present in almost every genome, which was different from previous studies (Girbau et al., 2015; Bruckner et al., 2020; Uljanovas et al., 2021), in which the presents of the virulence genes were identified by PCR. The lower detection ratio for these genes might be because of the genetic heterogeneity in primers’ target sequences.

Analysis of drug resistance genes showed that 10 acquired drug resistance genes were detected in ICDCAC48, all of which were located on a plasmid. And these resistance genes had a high degree of identity with those found in other bacteria, even in Gram-positive bacteria or their plasmids. Sequence analysis showed that *VEB-3* (beta-lactamase resistant gene) had 100% query cover and 99.89% sequence similarities with the *VEB-3* in *Enterobacter hormaechei* strain C45 plasmid pC45_002 (CP042553.1), *Citrobacter freundii* strain E33 plasmid pE33_002 (CP042519.1), *Klebsiella pneumonia* strain C51 plasmid pC51_003 (CP042484.1), and *Pseudomonas aeruginosa* strain SE5429 (CP054845.1). Here, we identified the presence of *VEB-3* in naturally occurring *Arcobacter* for the first time, which indicated that *VEB-3* might be exogenous. The transferable *optrA* gene encoded an ABC-F protein which conferred high levels of resistance to erythromycin, oxazolidinones, and chloramphenicol and has not so far been detected in *Arcobacter*. Plasmids carrying the *optrA* gene had the potential to be transferred between different *Enterococci* and between *Enterococci* and other Gram-positive bacteria (Wang et al., 2015). The *optrA* had 98.00% query cover and 99.58% sequence similarities with multiple plasmids in the *Enterococcus faecium* and *Staphylococcus sciuri* and was flanked by two copies of IS1380-like, suggesting that it may be transmitted by horizontal gene transfer. The *mexA*, *mexB*, and *oprM* were widely distributed in *Arcobacter*, indicating that they may be transmitted in different ways within the species. The *mefA*, *msrD*, and *ereB* were macrolide (erythromycin) antibiotic resistance genes that were initially reported in *Streptococcus*, and studies have shown that the presence of any of the *mefA*, *msrD*, and *ereB* could lead to high levels of resistance to macrolide antibiotics in *Streptococcus* (Arthur et al., 1986; Amezaga and McKenzie, 2006). The *ereB* has been reported in *E. coli* plasmids and was thought to be an exogenous gene of *E. coli* through plasmid transfer from *Streptococcus* (Arthur et al., 1986). These observations showed that certain strains with the potential to spread might represent a more significant hazard to humans than others. In particular, the MIC determination revealed that ICDCAC48 was resistant to erythromycin, azithromycin, ciprofloxacin, nalidixic acid, tetracycline, gentamicin, florfenicol, and telithromycin but only sensitive to streptomycin, chloramphenicol, and clindamycin, indicating that the drug-resistant phenotype of ICDCAC48 was conferred by drug-resistant genes on the plasmid. This was the first plasmid associated with drug resistance identified and reported in *A. cryaerophilus-like*. In addition, one-third (13/43) of Chinese strains contained at least two or two more tetracycline, aminoglycoside, and streptomycin resistance genes on the chromosome. However, the strains from other countries hardly contain any antibiotic resistance genes. Most strains’ resistance phenotype is perfectly correlated with the presence of a corresponding acquired resistance gene or mutation (Brooks et al., 2020). Therefore, these results reflected that the resistance situation of *A. cryaerophilus-like* in China might be more serious. Furthermore, the strains we collected showed that strains with a point mutation in the QRDR at position 254 of the *gyrA* gene were resistant to ciprofloxacin. By analogy with the Thr-Ile substitution in position 86 of the *gyrA* gene in *Campylobacter* ciprofloxacin-resistant strains, this substitution at Thr-85- Ile from *A. cryaerophilus-like* strains could be the cause of the quinolone resistance, which has been verified in *A. butzleri* (Ferreira et al., 2018; Isidro et al., 2020).

Plasmids are commonly present in diverse prokaryotes and have been reported in 9.9% of *A. butzleri* (Thomas, 2000; Toh et al., 2011; Douidah et al., 2014). Mobility of multidrug resistance plasmids in different environments was a major public health concern. The results of this study showed that the backbone of pCNAC48 was closely related to *Arcobacter* plasmids pM830MA, pATCC43158, and pAFAEC, which contained the same replicon *repA* and could not be assigned to any of the known incompatibility groups. Differences were also found in these plasmids, especially for pCNAC48. In addition to these plasmid replication and plasmid stability maintenance were found in most plasmids, two genomic islands, two prophages, antibiotic resistance genes, and several transposable genes were only identified in pCNAC48. Therefore, the presence of these traits in *A. cryaerophilus-like* pCNAC48 may represent a potential reservoir for wider gene transfer to other microorganisms. However, repeated attempts of conjugation assays failed to obtain transconjugants. The lack of *oriT*, relaxase, T4CP, and T4SS gene clusters might be responsible for the non-conjugative behavior of pCNAC48 compared to other transferable plasmids. Passaged cultures were performed in an antibiotic-free medium to verify the stability of the plasmid. No plasmid loss strain was obtained after 150 consecutive subcultures, which suggested that the plasmid was well stabilized in genetic evolution and difficult to lose.

A previous study found that resistance genes and non-conjugative plasmids could be mobilized by co-resident, conjugative plasmids, or IS (Di Sante et al., 2017). Five complete and nine partial IS elements were found in plasmid pCNAC48, several of these IS elements were identified in two or more *Arcobacter* species or showed high sequence similarity, suggesting that some members of the *Arcobacter* IS element suite had moved from species to species *via* horizontal gene transfer and may carry resistance genes for transfer. Two antibiotic-resistance gene islands were found in the plasmid with lengths of 7,950 and 25,137 bp and GC content of 38.23 and 32.39%, respectively. The GC content of antibiotic-resistance gene islands was significantly different from that of the chromosome of *A. cryaerophilus-like*, indicating insertion segments and antibiotic resistance genes in plasmid were exogenously acquired by *A. cryaerophilus-like via* horizontal gene transfer. Resistance genes also were flanked by transposons or IS in plasmid pCNAC48. The sequence alignment showed that *ISEnca1* and ISEnca1* were found in *Campylobacter* and *Arcobacter*, and *optrA* or *AAC(6′)-Ie-APH(2″)-Ia* were inserted between *ISEnca1* and ISEnca1*, which indicated resistance genes could be transferred by IS.

In this study, we first investigated the species classification for the strains isolated in China, and it was also a comprehensive description of the genetic characteristics of *A. cryaerophilus* based on genome features. The multiple drug resistance plasmid found in this study might explain the more resistant phenotypic characters in ICDCAC48.

## Conclusion

In conclusion, our results indicated that strains initially identified as *A. cryaerophilus* in this study should be referred to as *A. cryaerophilus-like* at the genome-level taxonomic, which was similar to *A. cryaerophilus* and cannot be distinguished by PCR at present. In this study, we obtained the genetic characteristics of *A. cryaerophilus* and *A. cryaerophilus-like* from different sources and exhibited a high intraspecies genomic diversity between the strains. A multidrug-resistant megaplasmid was identified and the characteristics of the plasmid were elucidated, contributing effectively to filling up the knowledge gap on this foodborne pathogen. The potential of plasmids to mediate horizontal transfer among *Arcobacter* and other species warrants further consideration by researchers interested in the risks to public health from these organisms.

## Data availability statement

The datasets presented in this study can be found in online repositories. The names of the repository/repositories and accession number(s) can be found in the article/Supplementary material.

## Author contributions

MZ designed the work and critically revised the manuscript. GZ analyzed, wrote, and revised the manuscript. MW and YG carried out the isolation and identification of the strains. HW, XC, ZS, and JZ revised the manuscript. All authors contributed to the article and approved the submitted version.

## Funding

This work was sponsored by the Sanming Project of Medicine in Shenzhen (SZSM201803081) and the National Key Research and Development Program of China (2021YFC2301000).

## Conflict of interest

The authors declare that the research was conducted in the absence of any commercial or financial relationships that could be construed as a potential conflict of interest.

## Publisher’s note

All claims expressed in this article are solely those of the authors and do not necessarily represent those of their affiliated organizations, or those of the publisher, the editors and the reviewers. Any product that may be evaluated in this article, or claim that may be made by its manufacturer, is not guaranteed or endorsed by the publisher.

## Footnotes

^1^ https://rast.nmpdr.org/

^2^ http://repeatmasker.org/RepeatMasker/

^3^ http://phaster.ca/

^4^ https://www.ncbi.nlm.nih.gov/

^5^ https://www.patricbrc.org/

^6^ https://lpsn.dsmz.de/

^7^ https://github.com/widdowquinn/pyani

^8^ https://github.com/tseemann/snippy

^9^ http://www.mgc.ac.cn/cgi-bin/VFs/v5/main.cgi?func=VFanalyzer

^10^ https://github.com/tseemann/abricate
